# The unique transcriptional response produced by concurrent estrogen and progesterone treatment in breast cancer cells results in upregulation of growth factor pathways and switching from a Luminal A to a Basal-like subtype

**DOI:** 10.1186/s12885-015-1819-3

**Published:** 2015-10-24

**Authors:** Eleanor F. Need, Luke A. Selth, Andrew P. Trotta, Damien A. Leach, Lauren Giorgio, Melissa A. O’Loughlin, Eric Smith, Peter G. Gill, Wendy V. Ingman, J. Dinny Graham, Grant Buchanan

**Affiliations:** 1 Cancer Biology Group, The Basil Hetzel Institute for Translational Health Research, School of Medicine, The University of Adelaide, DX465701, 28 Woodville Road, Woodville South, 5011 South Australia Australia; 2 Dame Roma Mitchell Cancer Research Laboratories and Adelaide Prostate Cancer Research Centre, The University of Adelaide, Adelaide, South Australia, Australia; 3 Freemasons Foundation Centre for Men’s Health, The University of Adelaide, Adelaide, South Australia Australia; 4 Present address: Icahn School of Medicine at Mount Sinai, Department of Oncological Sciences, Manhattan, New York USA; 5 Solid Cancer Regulation Research Group, The Basil Hetzel Institute for Translational Health Research Discipline of Surgery, The University of Adelaide, South Australia, Australia; 6 School of Medicine, Department of Surgery, The University of Adelaide, Adelaide, South Australia Australia; 7 School of Medicine at The Queen Elizabeth Hospital, University of Adelaide, South Australia, Australia; 8 Robinson Research Institute, University of Adelaide, South Australia, Australia; 9 Centre for Cancer Research, Westmead Millennium Institute, University of Sydney Medical School, Westmead, New South Wales 2145 Australia

**Keywords:** Progesterone receptor, Estrogen receptor, EGFR, Crosstalk, PAM50

## Abstract

**Background:**

In breast cancer, progesterone receptor (PR) positivity or abundance is positively associated with survival and treatment response. It was initially believed that PR was a useful diagnostic marker of estrogen receptor activity, but increasingly PR has been recognised to play an important biological role in breast homeostasis, carcinogenesis and metastasis. Although PR expression is almost exclusively observed in estrogen receptor positive tumors, few studies have investigated the cellular mechanisms of PR action in the context of ongoing estrogen signalling.

**Methods:**

In this study, we contrast PR function in estrogen pretreated ZR-75-1 breast cancer cells with vehicle treated ZR-75-1 and T-47D breast cancer cells using expression microarrays and chromatin immunoprecipitation-sequencing.

**Results:**

Estrogen cotreatment caused a dramatic increase in the number of genes regulated by progesterone in ZR-75-1 cells. In T-47D cells that have naturally high levels of PR, estrogen and progesterone cotreatment resulted in a reduction in the number of regulated genes in comparison to treatment with either hormone alone. At a genome level, estrogen pretreatment of ZR-75-1 cells led to a 10-fold increase in the number of PR DNA binding sites detected using ChIP-sequencing. Time course assessment of progesterone regulated genes in the context of estrogen pretreatment highlighted a series of important regulatory pathways, including those driven by epithelial growth factor receptor (EGFR). Importantly, progesterone applied to cells pretreated with estradiol resulted in switching of the PAM50-determined intrinsic breast cancer subtype from Luminal A to Basal-like, and increased the Oncotype DX® Unscaled Recurrence Score.

**Conclusion:**

Estrogen pretreatment of breast cancer cells increases PR steady state levels, resulting in an unequivocal progesterone response that upregulates key members of growth factor pathways. The transformative changes progesterone exerts on the breast cancer subtype suggest that these subtyping tools should be used with caution in premenopausal women.

**Electronic supplementary material:**

The online version of this article (doi:10.1186/s12885-015-1819-3) contains supplementary material, which is available to authorized users.

## Background

Breast cancer is the most commonly diagnosed invasive cancer in females [1] and is most often an estrogen (17β-estradiol) driven tumour [2, 3]. The primary cellular mediator of estrogen is the intracellular transcription factor estrogen receptor alpha (ERα), which is expressed in 75 % of early breast cancers [4]. ERα and PR positivity as assessed via immunohistochemistry of primary breast cancer is currently the gold standard indicator for hormonal therapy, applied either at the time of diagnosis or subsequent to surgical, chemotherapeutic and/or radiation management. While the molecular mechanisms and consequences of estrogen-mediated action have received considerable research attention, the molecular mechanisms of progesterone signalling have not been as widely reported. More recently PR is emerging as a key mediator of normal mammary gland development and tumorigenesis in mice, promoting mammary stem cell expansion and directing the immune microenvironment [5–10].

The majority of the cellular effects of progesterone are mediated by the progesterone receptor (PR), an intracellular transcription factor of which two isoforms exist, PR-A and PR-B. Because *PR* is an estrogen regulated gene, the expression of PR protein detected by immunohistochemistry as a diagnostic tool was found to discriminate between those most likely to respond to endocrine therapy, from those that will not [11, 12]. Indeed, expression of PR in breast cancer in the absence of ERα is rare (1.5 % of cases), and evidence suggests that such cases may represent false negatives for ERα staining upon re-analysis [13–16]. Nevertheless, PR appears to be more than a mere diagnostic indicator of estrogenic activity, as clinical studies have demonstrated it to be an independent biomarker of endocrine therapy response as well as a prognostic biomarker in postmenopausal breast cancers [12, 16–18]. Smaller studies in premenopausal women have found that tumours containing higher PR positivity had the best response to tamoxifen [19].

In premenopausal women, the physiological role of progesterone is inextricably linked to that of estrogen, with regards to production and secretion by the ovaries during the menstrual cycle. Increased production of estrogen by the maturing follicles ultimately results in ovulation, after which the corpus luteum produces and secretes progesterone. The secretion of progesterone in turn acts on the adrenal glands to stimulate a concomitant secondary, albeit smaller, peak of serum estrogen [20]. Evidence also suggests that the postmenopausal breast is capable of sequestering and/or synthesising progesterone and estrogen from circulating hormonal precursors [21–25]. Collectively, it appears most likely that PR is activated within a hormonal milieu that includes active estrogen signalling.

Genomic and functional studies of receptor action *in vitro* now provide unprecedented detail into the precise mechanics of ERα and, to a lesser extent, PR action in breast cancer cells. Those for PR have, however, been exclusively performed in the absence of exogenous estrogen [26–31]. Binding of estrogen by ERα and progesterone by PR results in association of the receptors with specific sites on chromatin. Receptor binding to DNA subsequently directs the recruitment of cofactors and associated coactivators and corepressors, resulting in modification of the local chromatin landscape and activation or repression of target genes. Indirect tethering of the receptors to chromatin has also been observed via interaction with DNA-bound factors such as AP-1, Stat3 and SP1 [27, 32, 33]. Despite the findings that PR expression is almost always accompanied by ERα expression [16], to date there are few reported studies investigating progesterone transcriptional signalling and PR binding in the context of estrogen-mediated signalling. Indeed, most studies of PR DNA binding have been performed in T-47D breast cancer cells that do not depend upon estrogen for PR expression [34]. In this report, we demonstrate a 10-fold induction in PR binding upon progesterone treatment in estrogen pre-treated versus non estrogen treated ZR-75-1 cells and demonstrate that progesterone and estrogen cotreatment drive a unique gene expression profile in ZR-75-1 that is distinct from treatment with either hormone alone, which includes up-regulation of signalling mediators of ErbB pathways. Estrogen and progesterone cotreatment cause significant changes to the predicted intrinsic breast cancer subtype, specifically to one that resembles more aggressive, therapy resistant disease.

## Methods

### Cell lines and culture

ZR-75-1, T-47D, MCF-7, MDA-MB-231, BT-20 and MDA-MB-453 cells were obtained from the American Type Culture Collection (Rockville, MD) and maintained in RPMI 1640 (Life Technologies, NSW, Australia) containing 10 % (ZR-75-1) or 5 % (T-47D, MCF-7) fetal bovine serum (FBS) (Sigma-Aldrich, NSW, Australia). All experiments were performed within 20 passages of supply from ATCC (Manassas, Virginia).

### Immunoblot analysis

ZR-75-1, T-47D, MCF-7, MDA-MB-231, BT-20 and MDA-MB-453 cells were seeded in 6 well plates at 5 × 10^5^ cells/well in phenol red free RPMI 1640 containing 5 to 10 % hormone stripped FBS (Sigma-Aldrich), in the proportions indicated for each cell type above. Hormone stripped treatment medium was supplemented with 10nM estrogen where indicated. After 72 h, medium was replaced with the indicated hormone treatment for the specified time. Cells were lysed, protein concentration assessed, electrophoresed and transferred to Hybond-C membrane as previously described [31]. Membranes were probed using AR-N20, PR-H190, ERα-HC20, CTSD-H75, FKBP5-H100 (Santa Cruz Biotechnology, CA), calnexin (CANX, Thermo Scientific, VIC, Australia), and anti-tubulin alpha (TUBA, Millipore, VIC, Australia) and detected as previously described [31].

### Microarray, RNA extraction and RT-qPCR

**Fig. 1:**
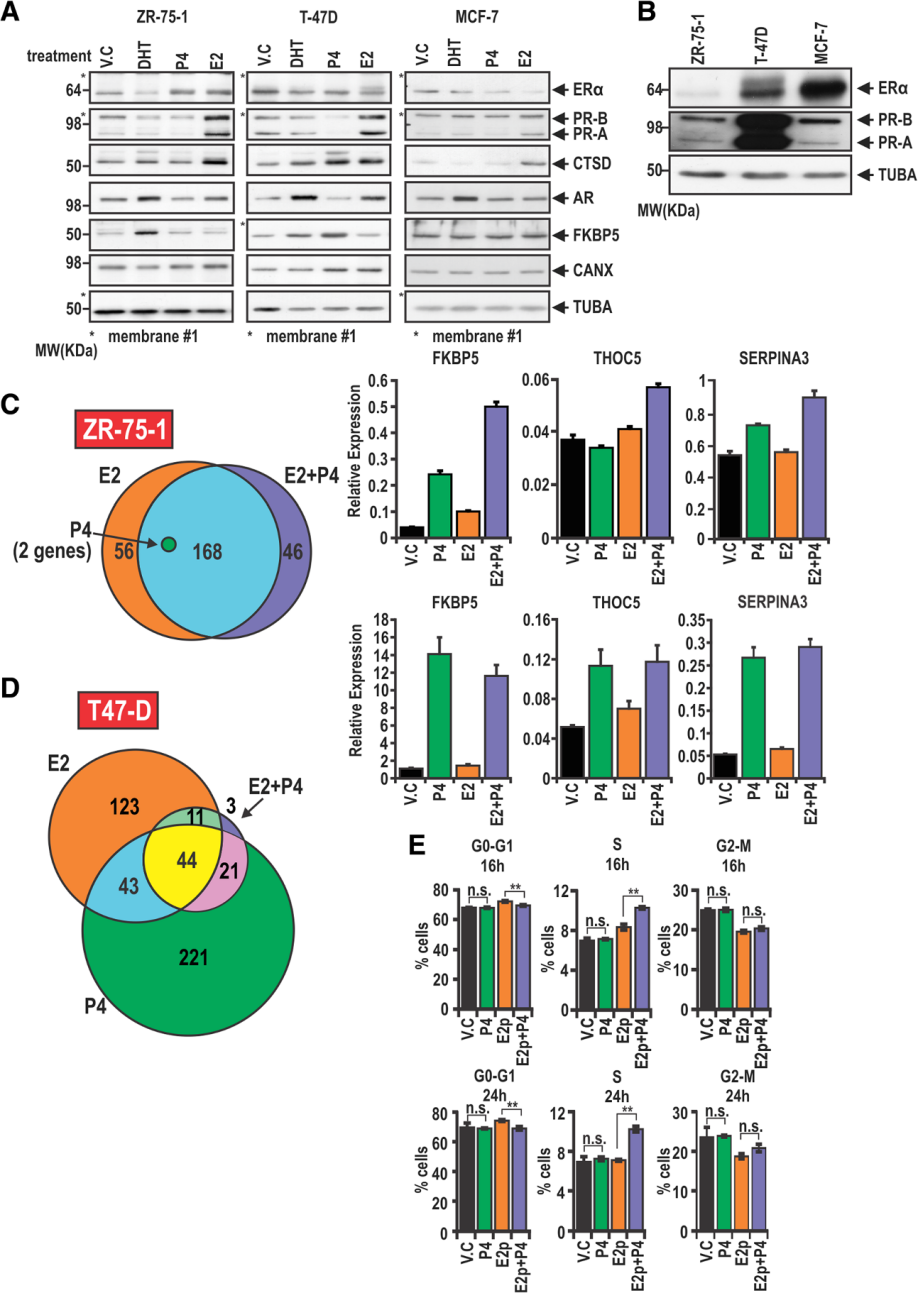
Estrogen and progesterone induce a unique transcriptomic response in ZR-75-1 and T-47D cells. **a** Protein steady state levels of ERα, PR-A, PR-B, androgen receptor (AR), androgen and progesterone regulated gene FKBP5 and estrogen regulated gene CTSD in ZR-75-1, T-47D and MCF-7 cells treated with ethanol (v.c.), 10nM DHT, 10nM PROG or 10nM estrogen for 16 h. TUBA and calnexin (CANX) were utilised as controls. Note that exposure time was different for each cell line and was optimised to visualise changes in response to hormone treatment. **b** Non hormone treated protein steady state levels of ERα, PR-A and PR-B in ZR-75-1, T-47D and MCF-7 cells treated with v.c. for 16 h. Alpha tubulin (TUBA) was utilised as a control. Exposure times were different from the blot presented in Fig. 1a. **c** Microarray analysis of the transcriptomic response of ZR-75-1 cells treated with ethanol (v.c.), 10 nM estrogen, 10 nM PROG, or cotreated with 10 nM estrogen and 10 nM PROG for 16 h. Euler diagram (left) demonstrates commonly regulated genes and those uniquely regulated by the hormonal cotreatment. Histograms (right) demonstrate validation of progesterone-regulated responses in independent samples. Expression presented relative to housekeeping gene *GAPDH* expression (**d**) Microarray analysis of the transcriptomic response of T-47D cells treated with ethanol (v.c.), 10 nM estrogen, 10 nM PROG, or cotreated with 10 nM estrogen and 10 nM PROG for 16 h. Euler diagram (left) demonstrates commonly regulated genes in response to each treatment. Histograms (right) demonstrate validation of progesterone-regulated responses in independent samples. **e** Cell cycle analysis of propidium iodide stained ZR-75-1 cells after treatment for 24 h with vehicle (V.C; ethanol), 10nM progesterone or pretreated for 72 h with 10nM estrogen (E2p), followed by 16 or 24 h of 10nM progesterone treatment (E2p + P4)

Cells were plated for 72 h in 6-well plates in phenol red-free RPMI 1640 containing 10 % hormone stripped FBS at 5 x 10^5^/ well, treated for 16 h with vehicle (ethanol; V.C), 10nM estrogen, 10 nM progesterone, 10 nM estrogen + 10nM progesterone, or for 72 h with 10nM estrogen (pretreated) with or without subsequent 10nM progesterone for 4, 8 or 16 h. RNA was extracted using RNeasy kit (Qiagen, VIC, Australia). The ZR-75-1 microarray results presented in Fig. 1 represent findings from quadruplicate samples randomly hybridised to Illumina HumanWG-6v3 chips (Australian Genome Research Facility, St Lucia, Australia). Raw transcript expression data was exported from Illumina BeadStudio software and analysed using the Bioconductor Limma package implemented in R [35], as previously described [31]. Briefly, we normalised array data using variance stabilisation normalisation [36], corrected the data with Combat [37], filtered to likely expressed transcripts (~24,000) and subjected the data to linear model fitting. Regulation compared to vehicle was accepted for an empirical Bayes moderated t-statistic incorporating Benjamini-Hochberg correction of ≤0.05. Microarrays in T-47D cells presented in Fig. 1 were performed in triplicate and were hybridised to Illumina HumanWG-6v2 chips (Genomics Core, Norris Comprehensive Cancer Centre, University of Southern California, USA). Raw transcript expression data was processed as described above, but subjected to two Combat corrections due to array batch effects. Samples for the ZR-75-1 time course microarray presented in Fig. 5 were generated in 5 × 10^5^ cells per well in 6 well plates in triplicate from ZR-75-1 cells treated with 72 h 10nM estrogen or vehicle, followed by 4, 8 or 16 h 10nM progesterone treatment. Hormone treatments were performed by overlaying the progesterone treatment on the existing media and the experiment was performed with reverse timing so all samples were collected at the same time point. Triplicate RNA samples were hybridised to human Gene 1.0 ST Affymetrix Arrays (Adelaide Microarray Centre, Adelaide, Australia). Raw CEL files were normalised, filtered for expressed transcripts (~23,875) and subjected to linear model fitting. Regulation compared to E2 pretreated samples was accepted for P4 treated samples for a Bayes moderated t-statistic with Benjamini-Hochberg correction of ≤0.0001, yielding a total of 2140 genes regulated at some point over the whole time course. Validation for all microarray results was performed on independent RNA samples by RT-qPCR using iQ SYBR Green Supermix (BioRad Life Science, NSW, Australia) on the CFX-96 PCR machine (Bio-Rad). Primer sequences are provided in Additional file 1. All microarray data is available online at NCBI (accessions GSE61538, GSE61368 and GSE62243). Pathway overrepresentation analysis was performed on differentially expressed genes using the comprehensive, publicly available InnateDB database, with hypergeometric testing and Benjamini-Hochberg correction for false discovery rates [38]. Clustering of microarray data was performed using the K-means clustering method, with 20 random starts in STEM, and a maximum output set to 8 model profiles [39].

### Cell cycle studies

ZR-75-1 cells were plated in 6 well plates in phenol red-free RPMI 1640 containing 10 % hormone stripped FBS and 10nM estrogen at 5 × 10^5^/ well for 72 h. Cells were then treated with 10nM progesterone or equivalent vehicle for 24 h. Cells were washed in PBS, harvested and fixed in ice cold 70 % ethanol. Fixed cells were incubated in 50 μg/ml propidium iodide (Sigma Aldrich), 40 μg/ml RNAse A (Life Technologies, NSW, Australia) and 0.1 % Tween20 (Sigma Aldrich) in PBS for 2 h in the dark. Cell cycle analysis was conducted on a FACSCanto II running DIVA software (BD Bioscience, NSW, Australia). DNA frequency histograms were obtained using FlowJo software (Treestar, Oregon, USA) using the Dean-Jett-Fox model. Results are representative of three independent experiments.

### Chromatin immunoprecipitation (ChIP) and ChIP-sequencing

ChIP and ChIP-sequencing was performed as previously described [31]. Briefly, ZR-75-1 and T-47D cells were plated for 72 h in phenol red-free RPMI 1640 containing 10 % hormone stripped FBS with 10nM estrogen or equivalent vehicle. After 72 h, medium was supplemented with the indicated hormone for 4 h. Immunoprecipitation was performed with PR-H190X or normal rabbit IgG antisera (Santa Cruz Biotechnology, CA). In total, 4 independent ChIP experiments were performed, each independently validated by RT-qPCR at an enhancer region of *FKBP5* and a nonspecific DNA region. Peaks were called and analysis was performed as described in [31]. Briefly, Genomic regions with a peak height of 3 (minimum of 3 independent 36 bp reads/site on a Illumina Genome Analyser II) were recorded using FindPeaks4 (Vancouver Short Read Analysis Package; http://vancouvershortr.sourceforge.net/) on human genome build 18 (hg18) and subsequent analysis was performed in R using custom algorithms as outlined in [31]. Bed files are provided as Additional files, and the primary data has been deposited at NCBI. Manipulation of intervals for analysing overlaps between different PR ChIP-seq datasets was performed in R, Galaxy [40] or BiSA [41]. The ChIP-seq datasets Conservation of binding sites amongst vertebrates was performed using the Cistrome Analysis Pipeline (http://cistrome.dfci.harvard.edu/ap). Regions of PR binding were annotated with respect to neighbouring genes using ChIPpeakAnno [42] and CisGenome [43]. High confidence sites were defined by our ability to empirically validate selected PR binding sites in independent samples (Additional file 2). To compare strength of PR binding at specific peak subsets, sequence tag libraries were generated and average tag density at the subsets was determined using the peak annotation function in HOMER v4.2 [44]. Novel sequence motifs that were present in PR binding regions statistically significantly more frequently than expected by random chance were identified using Gibbs Motif Sampling [45] or MEME [46]. Known sequence motifs in the JASPAR CORE vertebrata database [47] that were significantly enriched in the PR cistrome were identified using CisGenome, with default parameters [47, 48]. Fold enrichment and significance (Fisher’s exact test) of motif sequences were estimated compared with an equal number of 1-kb control regions with matched physical distribution.

## Results

### Shaping of the progesterone response by estrogen in breast cancer cells

To ascertain the most appropriate breast cancer cell line model to investigate the physiological progesterone response in the context of estrogen signalling, we assessed alterations in steady state protein levels of ERα, PR, androgen receptor (AR), Cathepsin D (CTSD) and FK506 binding protein 5 (FKBP5) in response to estrogen, progesterone and 5α-dihydrotestosterone (DHT) in a panel of breast cancer cell lines. Of the cell lines tested, only MCF7, T-47D and ZR-75-1 had detectable levels of both ERα and PR upon immunoblotting (Fig. 1a and Additional file 3). As the results in Fig. 1a were obtained with different exposure times, depending on the steady state level of the protein, we then compared the relative steady state levels of ERα and PR in MCF7, T-47D and ZR-75-1 cells and found that ZR-75-1 cells had the most equivalent detectable expression of all three receptors (Fig. 1b). Upon estrogen treatment, increased steady state levels of PR and CTSD were most dramatic in ZR-75-1 and T-47D cells, indicating activation of ERα. We observed that treatment of the cell lines with progesterone resulted in increased steady state levels of FKBP5 in T-47D cells but not in ZR-75-1 cells (Fig. 1a). This observation is not due to methodological artefacts as we were able to observe an increase in FKBP5 in ZR-75-1 cells in response to the androgen 5alpha-dihydrotestosterone (DHT).

To examine the potential regulatory effects of progesterone in the presence and absence of estrogen signalling, we performed microarray expression profiling of ZR-75-1 and T-47D cells following treatment with vehicle, estrogen, progesterone or both ligands in combination. Only 2 genes were regulated by progesterone alone in ZR-75-1 cells (*SERPINA3* and *SEPT4*; see Additional file 4). In contrast to these results, we were able to observe a small but consistent increase in FKBP5 expression upon RT-qPCR in ZR-75-1 cells in response to progesterone treatment, which was not detected using our cutoff criteria for differential expression on microarray (Fig. 1c; Benjamini-Hochberg corrected Bayesian moderated t-statistic *p <* 0.05). In agreement, this small increase in expression did not result in increased FKBP5 steady state levels upon progesterone treatment as observed by immunoblotting (Fig. 1c versus Fig. 1a). In contrast to the minimal effect of progesterone alone in ZR-75-1 cells, cotreatment with estrogen and progesterone resulted in significant regulation of 216 genes (Benjamini-Hochberg corrected Bayesian moderated t-statistic *p <* 0.05; Fig. 1c; see Additional file 4). Although 170 of these genes were also regulated upon estrogen treatment alone (78.7 %; Fig. 1c; see Additional file 4), 46 (21.3 %) were unique to the progesterone and estrogen cotreatment. In addition, cotreatment with progesterone resulted in the loss of regulation of 56 genes (25 %) observed with estrogen treatment alone (Fig. 1c; see Additional file 4). In T-47D cells in contrast, treatment with progesterone alone resulted in regulation of 329 genes, of which 87 (26 %) were also significantly regulated by estrogen alone (Fig. 1d; Additional file 5). Estrogen and progesterone cotreatment resulted in the loss of regulation of 24.9 % of estrogen responsive genes and 19.8 % of progesterone responsive genes. In contrast to ZR-75-1, only 3 genes were uniquely responsive to estrogen and progesterone cotreatment in T-47D cells (*GJB2*, *SSBP1* and *ZFP36*), and far fewer were regulated upon estrogen and progesterone cotreatment; 79 in T-47D, 216 in ZR-75-1 (Compare Fig. 1c to d). Results using independent sets of RNA samples reflect those findings, with candidate genes (*FKBP5, THOC5, SERPINA3*) showing significant upregulation in response to estrogen and progesterone cotreatment in ZR-75-1 cells, but no effect of estrogen and progesterone cotreatment in T-47D on these candidates in comparison to progesterone treatment alone (Fig. 1c). When the transcriptomic profiles of ZR-75-1 cells cotreated with progesterone plus estrogen were compared with T-47D treated with either progesterone only or estrogen plus progesterone, only 9.8 % (21/214) and 11 % (25/214) of genes were found to be in common. Collectively, these data indicate that the cotreatment of ZR-75-1 cells with estrogen sensitises the cells to progesterone and produces a unique transcriptional response that is distinct from the response mediated by estrogen or progesterone alone in either ZR-75-1 or T-47D cells.

Pathway analysis was performed separately on progesterone upregulated and down regulated genes in T-47D cells. Both of the gene lists were enriched for genes involved in cell cycle. In the upregulated gene list, transcriptional pathways were enriched, and pathways involved in DNA synthesis were significantly enriched in the downregulated gene list (see Additional file 6A and B). In estrogen and progesterone cotreated T-47D cells, fewer genes were regulated, but hormonal actions were over represented, such as glucocorticoid receptor regulation (see Additional file 6C and D). Enrichment of hormonal pathways was more evident in estrogen and progesterone treated ZR-75-1 cells, along with enrichment of genes involved in growth factor receptor signalling (Additional file 7A and B). These results suggest that estrogen and progesterone cotreatment in ZR-75-1 and T-47D cells produces a different transcriptomic response from progesterone alone in either cell type. Hence, the physiological effect of estrogen pretreatment on ZR-75-1 responsiveness to progesterone was assessed via cell cycle analysis using flow cytometry. Administration of progesterone to ZR-75-1 cells pretreated for 72 h with estrogen resulted in an small increase in the proportion of cells in the replicative S and G2M phases of the cell cycle, and fewer in the quiescent G0-G1 phases (Fig. 1e). This effect was not observed in cells treated with progesterone only and is consistent with those previously observed in other breast cancer cell lines and with the *in vivo* response in mice to estrogen and progesterone cotreatment [49, 50].

### Estrogen pretreatment increases PR genomic occupancy

**Fig. 2:**
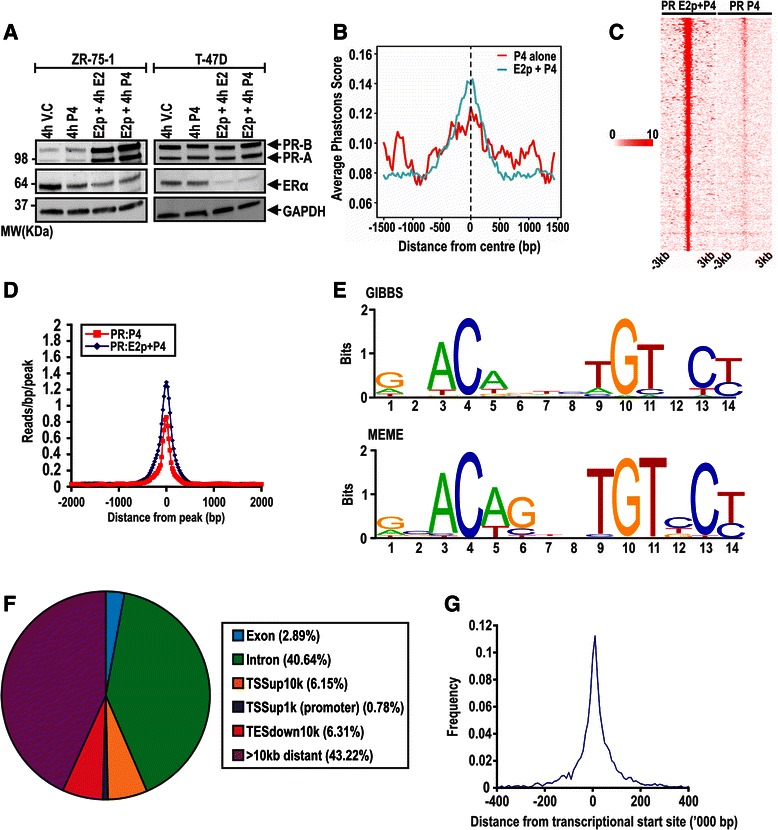
Estrogen pretreatment results in increased PR occupancy on DNA. **a** Steady state levels by immunoblotting of ERα, PR-A and PR-B in response to 4 h of 10nM progesterone treatment alone or 72 h 10nM estrogen treatment followed by 4 h 10nM progesterone treatment. **b** Conservation in the 475 progesterone alone PR binding sites versus the 4597 estrogen pretreated, progesterone PR binding sites in ZR-75-1 cells. **c** Relative strength of progesterone alone and estrogen pretreated, progesterone PR binding sites using peak annotation in HOMER. **d** The number of reads per peak are centred around the middle of the binding sites in both data sets. **e** *De novo* analysis of the estrogenpprogesterone PR dataset using both GIBBS and MEME revealed a PRE-like sequence as the most highly enriched motif. No PRE-like motif was found on *de novo* analysis of the progesterone alone dataset. (**f**) Distribution of the binding sites relative to the nearest TSS reveal a similar distribution to that of other studies [27, 29] and other receptors [31, 55]. **g** Binding sites were significantly enriched around the TSS of genes

To characterise PR action in the context of estrogen treatment, we performed PR ChIP-seq in ZR-75-1 cells treated with progesterone alone or after estrogen pretreatment of the cells with 72 h of 10nM estrogen. DNA pooled from 4 independently validated ChIP experiments (Additional file 8) was subjected to next-generation sequencing. After adjusting for input (see methods), 49,927 progesterone alone and 75,030 estrogen pretreated + progesterone binding sites were scored. Using these data, we identified 475 high confidence binding sites in the progesterone alone PR cistrome and 4597 high confidence estrogen pretreated + progesterone binding sites (Additional file 9; sites in .bed format). Only 31 of those high confidence sites were shared between the two cistromes, and had a much greater average peak height in comparison to sites not shared between the cistromes (Additional file 10A). Parallel analysis in T-47D cells validated these as likely PR binding sites, but there was little evidence of increased enrichment upon estrogen pretreatment (Additional file 10B). Western blotting revealed increased PR steady state levels in ZR-75-1 cells following estrogen pretreatment (Fig. 2a).

### The estrogen pretreated and progesterone alone PR binding sites are unique

Comparison of putative PR binding sites revealed a much greater sequence conservation amongst vertebrates for the progesterone treated, estrogen pretreated binding sites than the binding sites identified after treatment with progesterone alone, as well as a greater number of reads per peak (Fig. 2b-d). Using Gibbs Motif Sampling and MEME analysis approaches, the most highly enriched *de novo* motif in the estrogen pretreated PR cistrome resembled canonical PR binding sites, which were over-represented 3.24 and 3.69 respectively in comparison to the background genome average (Fig. 2e; *p =* <1 × 10^−200^, *p =* 1.49 × 10^−184^). Using these same tools, we were unable to identify a recognisable *de novo* hormone response element motif in the progesterone alone cistrome, perhaps partly due to the small number of sites interrogated. To identify factors that may regulate the association of PR with chromatin, we tested transcription factor binding motifs from the JASPAR CORE vertebrata database for enrichment in both PR cistromes (Additional file 11A and B). In the estrogen pretreated PR cistrome, the nine most highly enriched candidate motifs belonged to either steroid receptors or the forkhead family of transcription factors (most notably, FOXA1). Also enriched were motifs for transcriptional collaborators or tethering factors for steroid receptors (AP-1, STAT3, RUNX1, C/EBP [51, 52]). We also observed enrichment of binding sites for transcription factors implicated in cellular differentiation (TEAD1, ZEB1; HAND1; C/EBPa, SPI1; ZNF354C), consistent with a role for PR in this process in the breast [8]. In comparison, the progesterone alone cistrome was enriched for PR response elements, hormone response element half sites and several binding sites for the Forkhead (FOX) family. The transcriptional collaborators GATA2 and NKX3.1, which have been reported as transcriptional collaborators for PR and AR respectively [53, 54], were also significantly enriched in the progesterone alone PR binding sites.

**Fig. 3:**
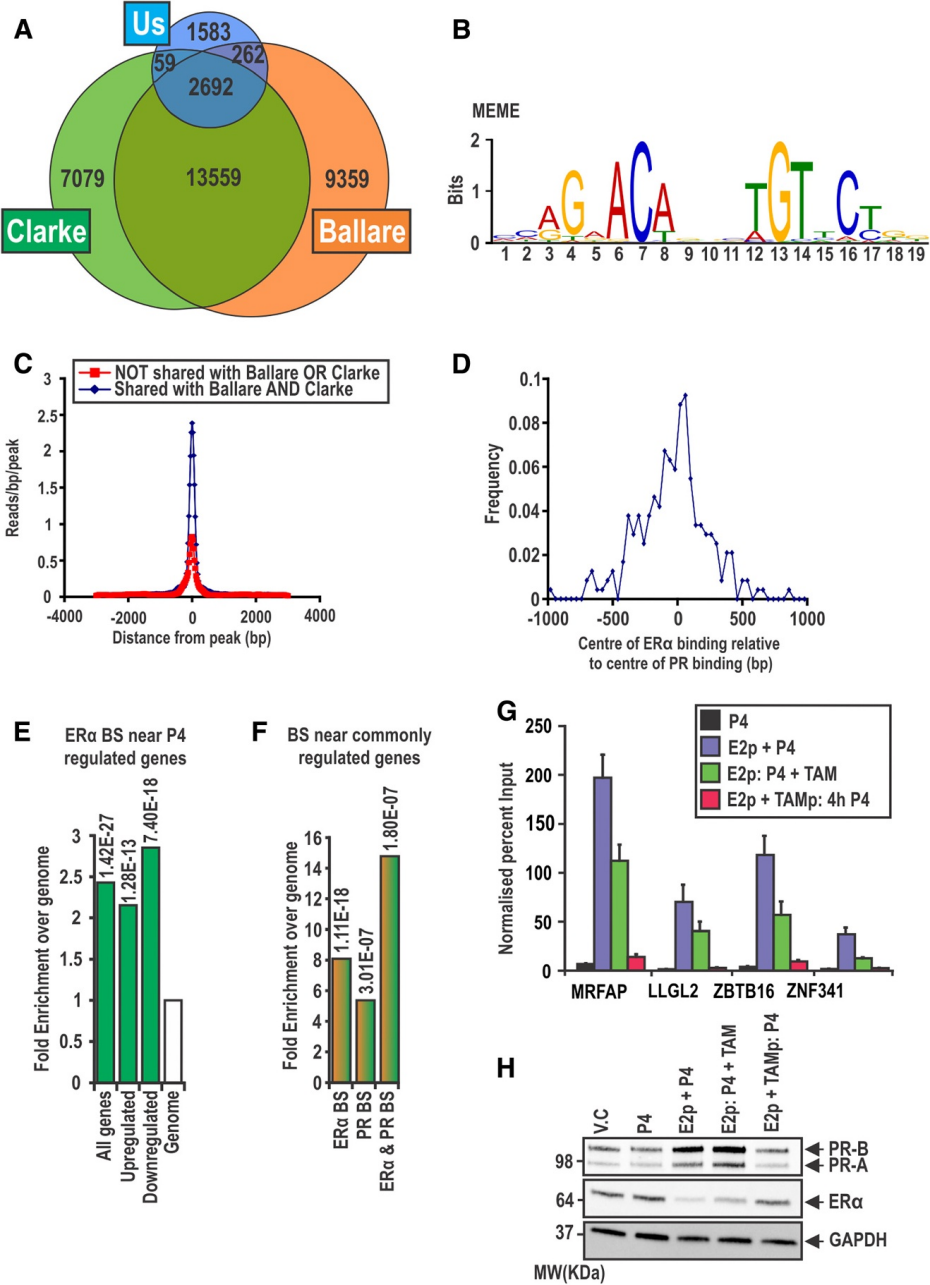
Overlap of PR binding sites with other cistromes, and assessment of the involvement of ERα in PR binding. **a** Assessment of overlap of our estrogen pretreated, progesterone PR binding site data with the more comprehensive Ballare and Clarke datasets [27, 29]. Clarke and our data was lifted over to hg18 using UCSC tools, and overlaps were calculated using BiSA. **b** *De novo* analysis of the 1836 overlapping binding regions between our dataset and those of Ballare and Graham reveals significant enrichment of a canonical HRE in these sites. **c** Comparison of the reads per peak between sites shared between all 3 data sets and the remaining 2761 sites reveal more reads per peak in the shared sites. **d** Alignment of binding sites shared between our previously published ERα binding sites and our estrogen pretreated, progesterone PR binding sites reveals close alignment between the centre of the binding sites. **e**, **f** Assessment of overlap (within 10 kb) between our estrogen pretreated progesterone treated PR binding sites and our previously published ERα binding sites and genes regulated by progesterone in estrogen pretreated cells. Numbers above each bar on the histograms represents the p value from Fishers exact test of the regions compared to an equal number of 1 kb control regions across the genome. **g** ZR-75-1 cells (1.2 × 10^7^ in 150 mm plates) were treated with vehicle or 10 nM estrogen for 72 h (pretreated; p) with or without 1 μM of the ERα specific antagonist Tamoxifen (TAM) and subsequently treated for 4 h with progesterone with or without 10 μM TAM. ChIP assays were performed using anti-PR and anti-IgG antibodies, and enrichment of the FKBP51 enhancer and nonspecific binding regions assessed by RT-qPCR. Data is representative of 2 repeated experiments, with the y axis representing the Normalised percent input to a nonspecific control region. **h** Steady state levels by immunoblotting with PR, ERα and loading control GAPDH of ZR-75-1 cells treated as described above in E

The estrogen pretreated, progesterone treated PR binding sites were distributed predominantly in introns and distal intergenic regions, with a moderate 13.24 % found within 10 kb of transcriptional start sites (TSS; Fig. 2f). Nonetheless, these regions were enriched around TSS in comparison to an equivalent number of random genomic regions (Fig. 2g). This distribution is similar to that reported by others for PR [27, 29] and for other steroid receptors such as ERα and AR [28, 31, 55]. For our estrogen pretreated + progesterone PR binding sites, 58-59 % overlap with two previously published PR cistromes from T-47D cells, providing good support for our empirically-based means of high confidence peak threshold estimation (Fig. 3a; [27, 29]). *De novo* scanning of the 2692 genomic regions shared between the 3 cistromes using MEME revealed significant enrichment of a motif that represents a canonical progesterone response element (Fig. 3b; E-value = 8.3 × 10^−41^). Moreover, the sites shared between the 3 cistromes had a significantly higher read density than those 1583 sites unique to our set of estrogen pretreated + progesterone PR binding sites (Fig. 3c). Together, these results suggest a core set of PR binding sites conserved between different breast cancer cell lines.

### Upregulation of PR steady state levels by estrogen is the primary mechanism of increased PR binding

As ERα and PR may interact on progesterone response elements to mediate transcriptional activation [56], we next assessed overlap between our previously published ERα cistrome in ZR-75-1 cells [31] with the estrogen pretreated PR cistrome generated here. Remarkably, that analysis suggested only 5.2 % overlap between PR and ERα binding sites in ZR-75-1 cells. Nevertheless, we did identify enrichment of ERα binding sites around (within 10kB) the transcriptional start site of genes regulated by estrogen and progesterone cotreatment in ZR-75-1 cells (*p =* 1.42 × 10^−27^; Fig. 3e), and enrichment of both ERα and PR binding sites near genes regulated by both estrogen alone, and by estrogen and progesterone cotreatment in these cells (*p =* 1.11 × 10^−18^; Fig. 3f). To elucidate, therefore, whether active ERα signalling is a requirement for PR DNA binding, we performed candidate PR ChIP in the presence of estrogen with or without the ERα antagonist TAM. As expected, we found that administration of TAM during estrogen pretreatment (that preceding progesterone treatment) compromised PR steady state levels and PR binding (Fig. 3g, h). When cells were pretreated with estrogen alone and then treated concurrently with TAM and progesterone, there was no effect on steady state PR levels (Fig. 3h), and only a small but consistent decrease in PR binding at a number of sites. Athough active ERα signalling may thus play a small role in strengthening PR binding at some sites, the most likely mechanism for the dramatic estrogen effect on the PR cistrome is via an increase in cellular PR levels.

**Fig. 4:**
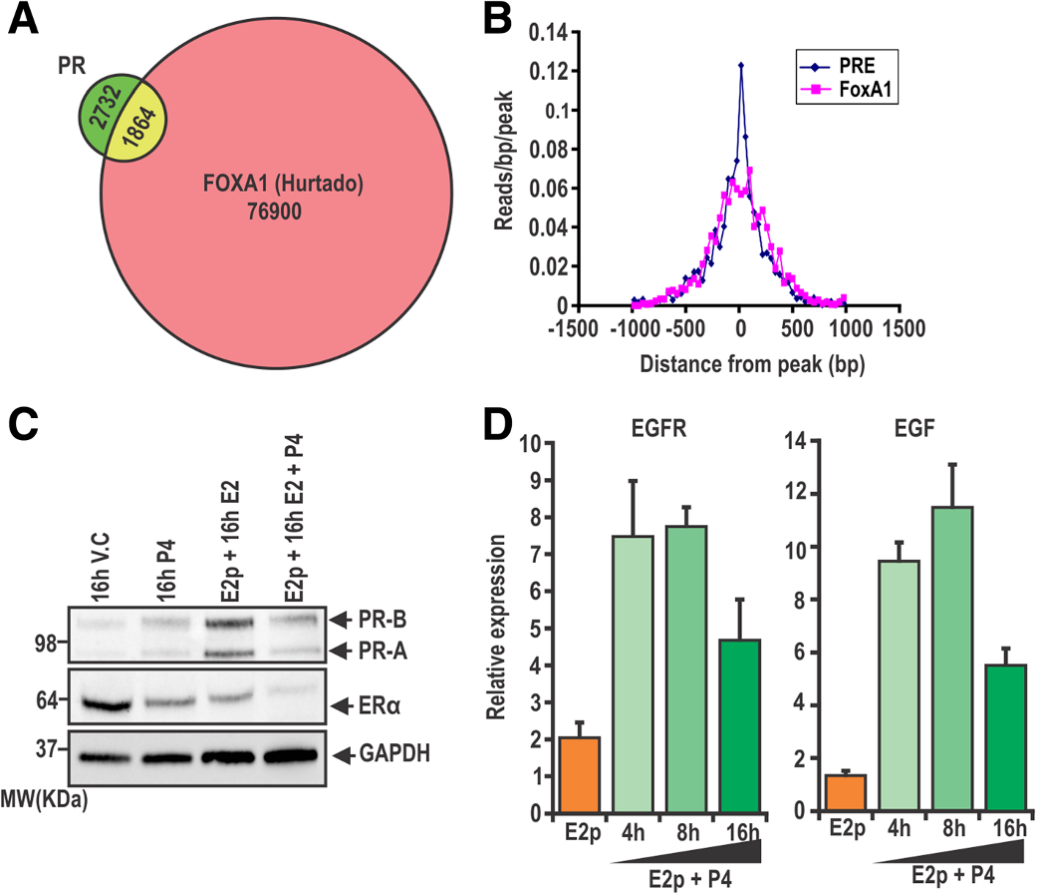
Peak-centred binding in sites shared by our PR and FOXA1 and upregulation of EGFR and EGF upon estrogen and progesterone treatment. **a** Overlap of FOXA1 binding sites identified in ZR-75-1 cells by Hurtado et al., with our estrogen pretreated, progesterone treated PR binding sites and our previously identified ERα binding sites. **b** Alignment of our estrogen pretreated, progesterone treated binding sites with the ZR-75-1 FOXA1 binding sites identified by Hurtado et al. [25]. **c** Steady state levels by immunoblotting of PR-A, PR-B, ERα and loading control GAPDH after 16 h 10nM progesterone or estrogen and progesterone treatment, with or without 72 h 10nM estrogen pretreatment (E2p). **d** Validation of up regulation of EGFR and EGF upon 72 h 10nM estrogen pretreatment followed by increasing times of incubation with 10nM progesterone in independently prepared RNA samples

An important collaborator involved in both ERα and PR DNA binding is FOXA1 [27, 55]. In this study, we found a 40.6 % overlap between our estrogen pretreated, progesterone treated PR binding sites and those previously published for FOXA1 in ZR-75-1 cells (Fig. 4a) [55]. Moreover, within these overlapping sites there was a strong concordance between peak centre and the location of predicted FOXA1 and PR response elements (Fig. 4b). This result reinforces the importance of FOXA1 in PR DNA binding, specifically in the context of estrogen treated cells.

### The estrogen pretreated progesterone transcriptomic response regulates growth factor signalling pathways

In order to comprehensively assess the transcriptional effects of progesterone in the context of active ERα signalling, we performed whole genome microarrays on RNA from estrogen pretreated ZR-75-1 cells subsequently treated with or without 4, 8 or 16 h of 10nM progesterone. As expected based on previous studies [57], the increased PR steady state levels seen with estrogen pretreatment were decreased following 16 h progesterone treatment (Fig. 4c). We identified 2140 genes that were significantly regulated over the progesterone time course in comparison to estrogen pretreated cells (*p <* 0.0001; Additional file 12). These results were validated on an independent RNA sample set (Additional file 13). Pathway analysis of this entire gene set revealed significant enrichment of genes involved in the EGFR pathway (NETPATH; *p =* 4.17 × 10^−10^), and in intracellular and chemokine signalling pathways such as MAPK and IL6 signalling (*p =* 0.008087 and *p =* 4.58 × 10^−5^, respectively; Additional file 14). To determine the early effects of progesterone treatment, we next assessed pathway enrichment for the 963 and 573 genes significantly up- or down-regulated respectively after 4 h of progesterone treatment. Both 4 h gene sets were significantly enriched in genes involved in the EGFR1 pathway (*p =* 0.00032 and *p =* 0.000836; Additional file 15A and B). Furthermore, we identified a significant overlap between genes reported to be transcriptionally regulated by EGFR (NETPATH ID#15908) and the entire 2140 estrogen pretreated, progesterone regulated gene set (43/154 genes = 28 %; Fishers exact test: *p =* 1.412 × 10^−13^). Significant upregulation of EGFR and EGF in response to progesterone in estrogen-pretreated cells was confirmed by RT-qPCR in an independent set of RNA samples (Fig. 4d), which is in line with previously published observations [58].

**Fig. 5:**
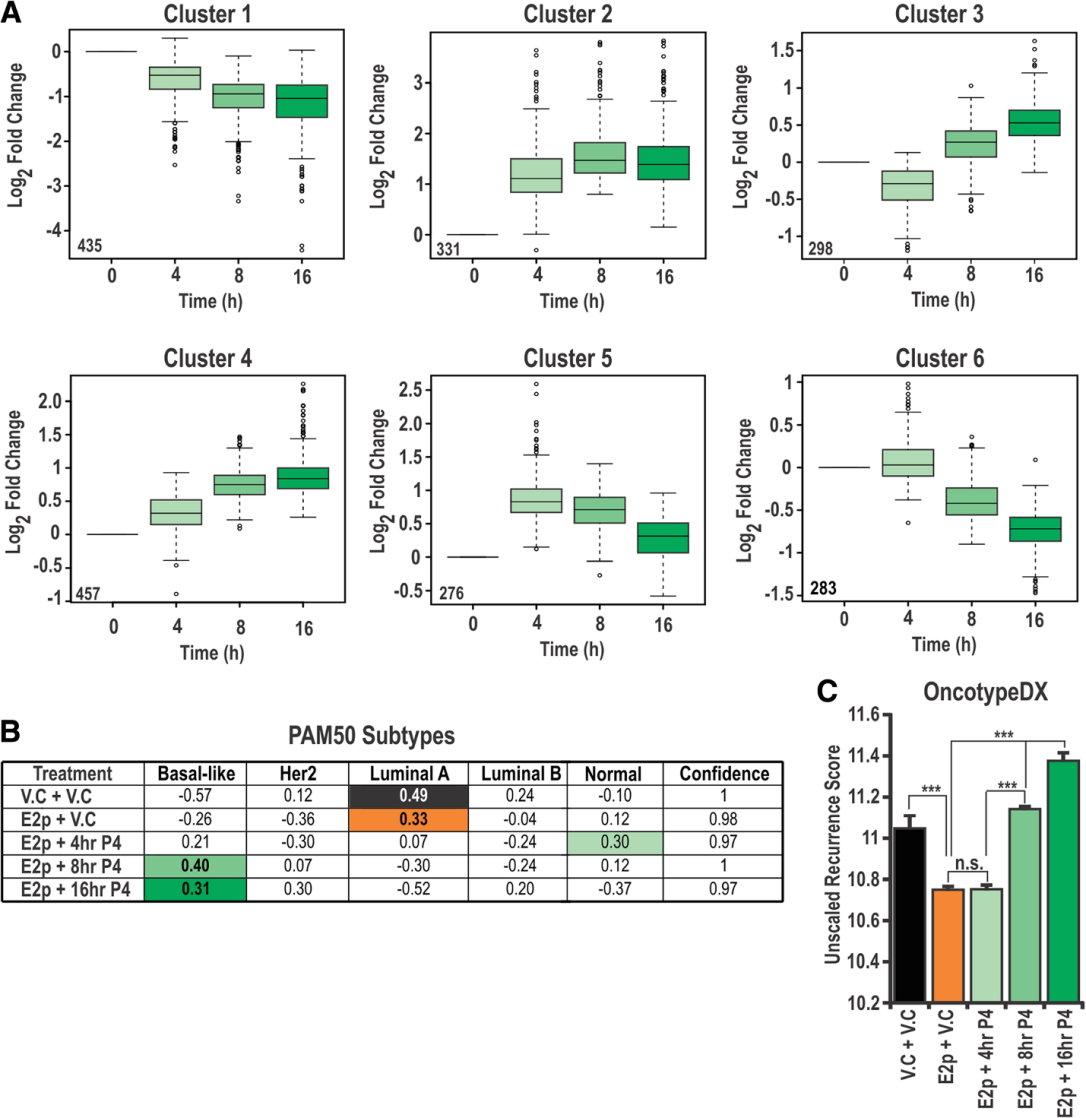
Assessment of the estrogen pretreated progesterone transcriptome reveals functionally associated clusters, and results in the ZR-75-1 cells switching from a Luminal A to a Basal subtype. **a** Cluster analysis reveals 6 functionally associated clusters. **b** PAM50 analysis utilising the microarray data using ZR-75-1 cells treated with 10nM estrogen for 72 h followed by vehicle treatment for 16 h (E2p + VC), or with 4, 8 or 16 h of 10nM progesterone treatment. Colours represent the closest centroid for each sample type. **c** Unscaled recurrence score (uRS) from Oncotype DX™ calculated from microarray data using the method described in [59]

To investigate the dynamics of progesterone transcriptional regulation in estrogen-pretreated cells, we undertook hierarchical clustering on the 2140 genes regulated over the progesterone time course. For that analysis, we reasoned there might be up to 8 general patterns, representing acute up or down regulation at one or more time points, or more consistent regulation in the same direction. Of the 8 unsupervised clusters generated, the pattern of regulation in Clusters 7 and 8 led us to collapse them into Clusters 1 and 2 respectively. Overall, there were two main trends of progesterone regulation. Acute effects were observed in Clusters 3 and 5, where time-dependent up or down regulation was observed followed by a return to baseline by 16 h. The remaining 4 clusters showed patterns of up or down regulation that were maintained over the 16 h time course (Fig. 5a). Pathway analysis of genes in Cluster 1 (chronically downregulated) revealed enrichment in nuclear receptor and steroid receptor regulation, and processes such as gland development and ovulation cycle (Additional file 16A). We reasoned that the downward pattern of regulation might indicate estrogen upregulated genes antagonized by co-treatment with progesterone. Indeed, 24.9 % (61/245) of our identified estrogen regulated genes (shown in Fig. 1c) were also found within Cluster 1. Cluster 2 genes, by contrast, were upregulated within 4 h of progesterone treatment and sustained there over the 16 h time course. This cluster was significantly enriched for genes involved in EGFR signalling, and for phosphorylation and kinase activity (Fig. 5a; Additional file 16B). Cluster 3 was acutely down regulated and enriched for genes involved in the EGFR1 pathway, as well as in cellular adhesion (Fig. 5a; Additional file 16C). The stepwise upregulation of genes in Cluster 4 represents enrichment of growth factor signalling (Fig. 5a; Additional file 16D), while acute upregulation and return to baseline in Cluster 5 is overrepresented by genes involved in Wnt and IL-6 signalling (Fig. 5a; Additional file 16E). Cluster 6 represents late downregulated genes, and is enriched for those involved in the TGFβ signalling pathway (Fig. 5a; Additional file 16F). Collectively, the above data identify progesterone, in the context of continuous estrogen exposure, as a regulator of a broad and unique transcriptional program distinct from that by either hormone alone. In the estrogen pretreated context, progesterone signalling regulates a number of important signalling pathways in breast cancer, perhaps most notably the ErbB signalling pathway.

### Treatment with progesterone modulates the intrinsic subtype status of estrogen pretreated breast cancer cells

To investigate further the impact of progesterone treatment on estrogen pretreated breast cancer cells, we applied the two common expression-based breast cancer phenotype tools, PAM50 and Oncotype DX® to our time-course expression array data. Both tools have either ERα signalling and/or growth factor receptor positivity at their core [59, 60]. Indeed, 31/50 (62 %) genes in the PAM50 algorithm [60] were significantly affected by progesterone treatment in estrogen pretreated cells (Additional file 17). Consistent with previous reports [61, 62], vehicle treated ZR-75-1 cells exhibit a predominantly ‘Luminal A’ subtype that was not altered in response to estrogen pretreatment (Fig. 5b). However, treatment with progesterone at 4, 8 and 16 h after estrogen pretreatment altered expression to such an extent that the closest PAM50 centroid changed to ‘Normal’ at 4 h and ‘Basal-like’ at 8 and 16 h (Fig. 5b). Assessment of the 21 gene algorithm contained within the Oncotype DX® test [59] indicated that estrogen pretreatment alone decreases the Unscaled Recurrence Score, whereas the addition of progesterone treatment results in a significant, time dependent increase in the Unscaled Recurrence Score (*p <* 0.0001; Fig. 5c).

## Discussion

In a recent meta-analysis, breast tumour subtyping via the Oncotype DX® platform was found to guide clinical decision making regarding the use of adjuvant chemotherapy in 34 % of early breast cancer cases [63, 64]. Moreover, the St Gallen International Expert Consensus found that microarray-based intrinsic subtype classification of breast cancers is an important guide for chemotherapy use in ERα positive, HER-2 negative disease [65]. That panel did however recognise the potential prohibitive cost of wide-spread multigene expression analysis, and instead propositioned immunohistochemical surrogates such as dichotomising ERα positive breast cancer cases on the basis of PR and Ki67 positivity thresholds and HER2 status, even though such measures have been found to be less accurate [17, 60, 65]. Despite increased recognition and utility of subtype classification in the clinical setting, the factors or conditions that drive individual tumours into classifiable subtypes are currently unknown. Even though this study was conducted in breast cancer cell lines, the findings of this study suggest that exposure to hormones may alter the transcript profile of breast cancer cells sufficiently to change their classification by multi-gene algorithms. Specifically, we found that estrogen pretreated breast cancer cells exhibit a Luminal A subtype, which switches to a Basal-like subtype upon combined estrogen and progesterone treatment. In support of steroid-induced effects on intrinsic subtypes, the incidence of Basal-like tumours decreases significantly with age, from 44 % in premenopausal aged patients (21–39 years) to just 9 % in patients aged 70–93, who exhibit lower, more static serum levels of progesterone and estrogen [66]. Indeed, the expression of PR and other key estrogen regulated genes in breast tissue from postmenopausal women is positively associated with serum estrogen levels [67]. In the pre-menopausal setting, a study of estrogen regulated genes throughout the menstrual cycle in early breast cancer samples demonstrated a significant increase in PR transcript and protein levels during follicular and luteal phases (days 7–26), corresponding with higher known circulating estrogens [68]. Likewise, the expression of PR, a PR regulated gene *RANKL*, and an ERα regulated gene, *TFF1* are all significantly higher in premenopausal in comparison to postmenopausal women [69]. Recent studies demonstrate intra-individual variability in multigene signature scores between fine needle biopsies and resection specimens [70]. Finally, PR abundance may decrease upon activation by progesterone treatment, adding to the complexity of using PR abundance as a surrogate for intrinsic subtype status [57]. While the study reported herein is provocative, these findings require careful validation in premenopausal breast cancer patients. In the meantime, these data suggest that careful consideration be given to the menopausal status of women, and the concentration of circulating estrogen and progesterone at the time of tumour collection, if RNA-based subtyping tools, and perhaps their immunohistochemical surrogates are to be used in clinical decision making.

The potential for plasticity between the intrinsic subtypes of breast cancer has not been widely investigated. From a clinicopathological perspective, nearly 70 % of Basal-like tumours and just 3 % of Luminal A tumours have a triple negative phenotype (ERα and PR negative and no HER2/neu overexpression) [71], and 65 % of ERα negative/PR positive tumours exhibit a Basal-like PAM50 subtype [63–65]. Furthermore, tumours arising in younger women have significantly lower ERα and PR expression, but higher HER-2 and EGFR expression [72], and in Basal-like breast cancers and breast tumours in younger women, the level and expression of EGFR is an adverse prognostic factor [72, 73]. While the studies contained herein are preclinical in nature, we describe that combinatorial estrogen and progesterone treatment result in upregulation of several key members of the EGFR signalling pathway. If this relationship is verified in premenopausal and postmenopausal breast cancers, it is possible that subtyping tools developed predominantly from postmenopausal women may be particularly prone to menstrual cycle-induced plasticity or hormone-driven artefacts.

In ERα positive breast cancers, PR positivity is indicative of a more favourable response to endocrine therapy [16], but does not distinguish between a clinical response to tamoxifen or aromatase inhibitors [18, 74]. Nonetheless, the percent and intensity of breast cancer cells positive for PR protein by immunohistochemistry is positively correlated with time to recurrence in both tamoxifen and anastrozole treated patients, and Luminal A type breast cancers containing more than 20 % PR positive cells have a better prognosis than those with less than 20 %, independent of endocrine therapy [59]. Thus, while abundance of PR provides prognostic information beyond ERα positivity, the important question is whether this derives from the intrinsic biological activity of PR, or is purely due to PR acting as a marker of the extent of tumour cell ERα activity or responsiveness. The intrinsic biological role of PR has been difficult to study in breast cancers precisely because of its dependent relationship on ERα, and the concordance between levels of ERα and PR in breast cancers [12, 34, 75]. We show here that PR action is dependent on the hormonal context, with concurrent estrogen treatment producing a unique transcriptomic response to progesterone. Combined with our finding that the master regulator of a progesterone response in breast cancer cells appears to be estrogen, which regulates PR abundance, thereby permitting PR DNA binding, our findings suggest that the actions of estrogen and progesterone are inextricably linked. Interestingly, the ancestral vertebrate steroid receptor was a receptor that preferentially bound estrogens, with the progesterone receptor the second steroid receptor to evolve [76, 77]. Hence, the estrogen and progesterone receptors have the longest coexistence in relation to the other steroid receptors, so perhaps it is not surprising that a complex functional regulatory relationship exists between them, where ERα-mediated upregulation of PR abundance permits activity in response to progesterone, and PR in turn, regulates a subset of ERα actions [78, 79]. Mechanistically, we anticipate that a large part of the unique response observed here is the sensitization to progesterone mediated by upregulation of PR by estrogen, resulting in a combined estrogenic/progestogenic response. Given that we observed a large overlap in binding sites between ZR-75-1 cells cotreated with estrogen and progesterone and those previously reported in T-47D cells treated solely with progesterone, alternative binding of the PR induced by estrogen treatment is unlikely to be the sole cause of the unique estrogen and progesterone transcriptome observed here. One possibility is that estrogen treatment may cause differential regulation of transcriptional collaborators, such as FOXA1. While further studies will determine the precise mechanism, we propose that the counter-regulation of approximately one quarter of estrogen responsive genes upon progesterone treatment, and upregulation of growth factor receptor pathways, may together contribute to the unique transcriptome observed here.

HER2 and/or EGFR overexpression is a cause of endocrine resistance, and ER positivity has been shown to decrease the effectiveness of HER2 targeting agents and provide a potential avenue for resistance to HER2-targeted therapies [55, 80–86]. Many molecular and clinical studies suggest that HER2 and hormone receptor positive breast cancers have the ability to switch between hormonal-driven and ErbB-driven signalling, with this switch mediating therapeutic resistance. This suggests that each of these two pathways are sufficient to propagate cancer cell growth, with the mechanistic switch perhaps partly being explained in terms of estrogen-ERα complexes or tamoxifen-ERα complexes repressing HER2 transcription [55]. Here, our data suggest that PR may collaborate in the relationship or interplay between hormonal and ErbB signalling. While only in a single breast cancer cell line, we demonstrate the potential for progesterone to activate EGFR signalling, consistent with progesterone potentiation of EGF responses in ZR-75-1 cells [48]. In early breast cancers moreover, those carrying a gene signature representing activity of hyperphosphorylated PR were found to have higher prevalence of HER2 positivity and distal metastasis [60]. Together, these findings firmly position PR as much more than a marker of ERα action in breast cancer, and our observations that both estrogen and progesterone play a role in the upregulation of growth factor receptor pathways suggest that PR targeting should be considered more closely as a partner in currently employed endocrine and ErbB-targeted therapies.

## Conclusions

We demonstrate hormone-induced plasticity of subtype status in breast cancer cells, confounding the notion of an inherent intrinsic subtype. This is pertinent given the expanding role of subtyping tools in the clinical setting, and these results are particularly relevant for the use of these subtypes or their surrogates in premenopausal women. In addition, our data suggests that PR may act as a mediator between ErbB-driven and hormonal-driven cancer cell growth, and could represent a mechanism of hormonal treatment resistance that could be targeted using currently available therapies.

## Availability of supporting data

Microarray data underpinning data in Fig. 1 is available online at the NCBI GEO database at http://www.ncbi.nlm.nih.gov/geo; accession 61368 for ZR-75-1 studies and accession 62243 for T-47D cell studies. Microarray data underpinning Fig. 5 is available online at the NCBI GEO database at http://www.ncbi.nlm.nih.gov/geo; accession GSE61538. Sequence data is available online at the NCBI Sequence Read Archives at http://www.ncbi.nlm.nih.gov/sra; accession PRJNA252531.

## Additional files

Additional file 1: **Sequences of Primers used in this report.** (XLS 11 kb)
Additional file 2: **Validation of binding sites by peak height in independent samples.**ZR-75-1 cells were plated, treated, ChIP and subjected to RT-qPCR as described in materials and methods. (XLS 1611 kb)
Additional file 3: **Assessment of receptor negative cells for E2-mediated PR upregulation.** Relative steady state levels of ERα, PR-A, PR-B, AR and the ERα regulated gene CTSD and PR regulated gene FKBP5 in the ERα and PR negative breast cancer cells BT-20, MDA-MB-231 and MDA-MB-453 treated for 16 h with ethanol (v.c.), 10nM DHT, 10nM P4 and 10nM E2. (XLS 760 kb)
Additional file 4: **Genes significantly regulated by hormonal treatments in comparison to VC treatment in ZR-75-1 cells.** (XLS 356 kb)
Additional file 5: **Genes significantly regulated by hormonal treatments in comparison to VC in T-47D cells.** (XLS 233 kb)
Additional file 6: **Significantly enriched pathways in response to hormonal treatment in T-47D cells.** (XLS 41 kb)
Additional file 7: **Significantly enriched pathways in response to hormonal treatments in ZR-75-1 cells.** (XLS 10 kb)
Additional file 8: Figure S2. Validation of the 4 independent ChIP assays that were pooled for ChIP-sequencing. (XLS 884 kb)
Additional file 9: **BED files of the sites found to be enriched ZR-75-1 cells in E2 pretreated + P4 cells and in cells treated with P4 treatment alone.** (XLS 510 kb)
Additional file 10: **E2 priming is not critical for PR binding in T-47D cells.** (XLS 169 kb)
Additional file 11: **Motif enrichment in the ZR-75-1 ChIP-sequencing datasets.** (XLS 11 kb)
Additional file 12: **Complete list of 2140 genes found to be significantly upregulated upon a timecourse of P4 treatment in E2 pretreated cells.** (XLS 974 kb)
Additional file 13: **Validation of E2 primed P4 regulated genes in an independent sample set.** ZR75-1 cells (5 x 105 cells per well in 6 well plates) were treated in triplicate with 72 h 10nM E2 followed by 4, 8 or 16 h 10nM P4 treatment. Expression relative to housekeeping gene GAPDH expression. (XLS 726 kb)
Additional file 14: **Significantly enriched pathways in response to a timecourse of P4 treatment in E2 pretreated ZR-75-1 cells.** (XLS 26 kb)
Additional file 15: **SIgnificantly enriched pathways in response to 4 hours of P4 treatment in E2 pretreated cells.** (XLS 22 kb)
Additional file 16: **SIgnificantly enriched pathways by gene response cluster.** (XLS 40 kb)
Additional file 17: **Assessment of the Estrogen primed, progesterone regulation of PAM50 genes.** (XLS 386 kb)

## Abbreviations

AR: Androgen receptor
ChIP-seq: Chromatin immunoprecipitation-sequencing
CTSD: Cathepsin D
DHT: Dihydrotestosterone
estrogen: E2
E2p: Estrogen pretreated
EGFR/ERBB1: Epidermal growth factor receptor
ERα: Estrogen receptor alpha
ErbB: Erythroblastic Leukemia Viral Oncogene Homolog
FKBP5: FK506-binding protein 51
GR: Glucocorticoid receptor
HT: Hormonal therapy
PR: Progesterone receptor
RT-qPCR: Real time quantitative polymerase chain reaction
TAM: Tamoxifen
